# The Role of Melatonin on NLRP3 Inflammasome Activation in Diseases

**DOI:** 10.3390/antiox10071020

**Published:** 2021-06-24

**Authors:** Burak Ibrahim Arioz, Emre Tarakcioglu, Melis Olcum, Sermin Genc

**Affiliations:** 1 Izmir Biomedicine and Genome Center, Dokuz Eylul University Health Campus, 35340 Izmir, Turkey; burak.arioz@msfr.ibg.edu.tr (B.I.A.); emre.tarakcioglu@msfr.ibg.edu.tr (E.T.); melis.olcum@ibg.edu.tr (M.O.); 2 Izmir International Biomedicine and Genome Institute, Dokuz Eylul University, 35340 Izmir, Turkey; 3 Department of Neuroscience, Health Science Institute, Dokuz Eylul University, 35340 Izmir, Turkey

**Keywords:** melatonin, NLRP3 inflammasome, disease

## Abstract

NLRP3 inflammasome is a part of the innate immune system and responsible for the rapid identification and eradication of pathogenic microbes, metabolic stress products, reactive oxygen species, and other exogenous agents. NLRP3 inflammasome is overactivated in several neurodegenerative, cardiac, pulmonary, and metabolic diseases. Therefore, suppression of inflammasome activation is of utmost clinical importance. Melatonin is a ubiquitous hormone mainly produced in the pineal gland with circadian rhythm regulatory, antioxidant, and immunomodulatory functions. Melatonin is a natural product and safer than most chemicals to use for medicinal purposes. Many in vitro and in vivo studies have proved that melatonin alleviates NLRP3 inflammasome activity via various intracellular signaling pathways. In this review, the effect of melatonin on the NLRP3 inflammasome in the context of diseases will be discussed.

## 1. Introduction

The NLRP3 inflammasome is a crucial step in innate immune responses and contributes to immune pathogenesis of several diseases including neurodegenerative, cardiac, pulmonary, gastrointestinal, and metabolic diseases. Therefore, inhibition of NLRP3 inflammasome activation is a novel therapeutic target for inflammation-related disorders. In recent years, many natural or synthetic molecules targeting NLRP3 have been evaluated for this purpose. Melatonin is one of the best candidate agents due to it being a natural and endogenous molecule. Here, we reviewed the suppressive effect of melatonin on the NLRP3 inflammasome in various in vitro and in vivo models of diseases and injuries. We performed a literature search in the National Library of Medicine’s PubMed and PubMed Central. The following queries were utilized: “melatonin AND NLRP3” in PubMed and “(melatonin[Body-All Words] AND NLRP3[Body-All Words])” in PubMed Central. We identified a total of 411 (48 from PubMed and 363 from PubMed Central) articles. After excluding duplicates and inaccessible articles, and applying our inclusion criteria, 38 articles were selected for our review. In our review, the following criteria were applied for article inclusion: (1) using melatonin treatment in vivo or in vitro; (2) reporting any protective effect of melatonin against NLRP3 inflammasome (we also included an article that investigates a synthetic derivative of melatonin); (3) full-text research articles in the English language. Others were excluded unless specified otherwise.

## 2. Melatonin

Melatonin (*N*-acetyl-5-methoxy tryptamine) is a hormone produced from L-tryptophan via a chain of enzymatic reactions, mostly in the pineal gland [1,2]. One of the well-recognized functions of melatonin is circadian rhythm regulation. Additionally, anti-inflammatory, cytoprotective, free radical scavenging, antioxidative, anti-cancer, anti-aging, and immunomodulatory effects are also reported [3,4,5]. Two different membrane receptors for melatonin binding have been identified until today: Melatonin may bind to high-affinity MT1 (Mel1a) and MT2 (Mel1b) receptors which belong to the G protein-coupled receptor (GPCR) family [6]. Other than cell membrane receptors, melatonin can bind to the intracellular MT3 (Mel1c) receptor [7], calmodulin [8], calreticulin [9], and retinoic acid receptor superfamily (retinoid Z receptors/retinoid orphan receptors (RZR/ROR)): RORα splicing variants (RORα1, RORα2, RORα3, RZRα), RZRβ, and RORγ [10,11]. In addition to interactions with all receptors, melatonin has the ability to pass through membranes due to its amphiphilicity; so, it may react with molecules in cells without the help of receptors, termed as non-receptor mediated actions [12]. A complete overview of anti-inflammatory actions of melatonin can be found in previous articles [13,14]. In this review, we focus on the NLR family pyrin domain containing 3 (NLRP3) inflammasome, and we summarize the recent studies on the effects of melatonin on the NLRP3 inflammasome activation in a variety of diseases and possible intracellular mechanisms of these effects.

## 3. NLRP3 Inflammasome and Regulation

The inflammatory response is a crucial response for survival, governed by pro-inflammatory cytokines and chemokines. The innate immune system is the first to encounter pathogens and threats to the organism and respond immediately. Hyperactivation or abnormal activation of the inflammation response leads to the high concentration of cytotoxic molecules, in turn leading to tissue damage [15]. Inflammasomes are vital contributors to innate immunity. These are cytosolic multiprotein complexes composed of a sensor protein (an AIM-like receptor or NOD-like receptor (NLR)), an adaptor protein, and an effector protein. When the inflammasomes are activated with a danger signal, cytokine secretion is triggered to clear pathogens or damaged cells [16]. The NLR family of cytosolic pattern recognition receptors are vital in recognizing intracellular bacterial breakdown products and starting an innate immunity cascade. It has several members such as NLRP1, NLRP2, NLRP3, NLRC4, NLRP7, NLRP6, and NLRP9b [17,18,19,20,21,22]. NLR family receptors contain a C-terminal leucine-rich repeat ligand sensing region (LRR), a NOD domain, and an N-terminal signaling module. This module may be a caspase recruitment domain (CARD), a pyrin domain (PYD), or a baculovirus inhibitor of apoptosis repeat [16].

The most extensively studied inflammasome complex is NLRP3, and it has a vast repertoire of recognition, enabling easy activation of immunity and pathogen clearance. The complex formation is activated via internal and/or external factors; the primary factors being pathogen-associated molecular patterns (PAMPs), danger-associated molecular patterns (DAMPs), reactive oxygen species (ROS), ion fluxes, lysosomal destabilization, ATP, and peptide aggregates [23]. Once activated on its LRR domain, NLRP3 recruits an adaptor protein called ASC (apoptosis-associated speck-like protein containing a CARD) on its pyrin domain. ASC oligomerizes with other recruited ASCs to form what is termed ASC specks, and in return, recruits pro-caspase-1 on its CARD containing end and triggers caspase-1 activation [23,24,25]. Pro-caspase-1 is an auto-proteolytic enzyme, and the proximity induced by ASC specks thus generates caspase-1. After activation, the complex triggers inflammatory cytokine secretion which ultimately leads to pyroptosis [26]. Canonical activation of the NLRP3 inflammasome requires two consecutive signals: priming and activation. The priming signal is initiated via TLR receptors by PAMPs such as LPS and leads to transcription of inflammatory cytokine genes through activation and nuclear translocation of NF-κB. The activation signal is the stimulation of NLRP3 by the various factors it recognizes, which eventually results in pro-caspase-1 cleavage [23]. Cleavage of pro-caspase-1 results in two enzymatically active caspase-1 subunits: p10 and p20 [27]. Active caspase-1 cleaves pro-inflammatory cytokines IL-1β, IL-18, and the protein Gasdermin D into their mature forms. Mature Gasdermin D forms pores on the cellular membrane, resulting in pyroptotic cell death and release of cytokines [28]. IL-1β and IL-18 amplify NLR-mediated inflammation and facilitate infiltration of immune cells [15].

Strictly regulated NLRP3 inflammasome is crucial. Post-transcriptional, post-translational, and negative regulation mechanisms enable tightly controlled NLRP3 activation [16]. At the post-translational level, the deubiquitinating enzyme BRCA1/BRCA2-containing complex subunit 3 (BRCC3) instigates NLRP3 activation [29]. On the other hand, F-box L2 [30], TRIM31 [31], and MARCH7 [32] were shown to alleviate NLRP3 inflammasome activation by ubiquitination of NLRP3. Protein tyrosine phosphatase non-receptor 22 [33] and protein phosphatase 2A [34] augment NLRP3 inflammasome by dephosphorylation, while Jun N-terminal kinase [35] and protein kinase D [36] augment NLRP3 inflammasome by phosphorylation of NLRP3. 

Thioredoxin-interacting protein (TXNIP) regulates intracellular redox balance by inhibiting important antioxidant proteins called thioredoxins and, upon amplification of intracellular ROS levels, TXNIP dissociates from thioredoxins and induces NLRP3 activation by binding to it [37]. Endoplasmic reticulum (ER) stress is another known inducer of NLRP3 activation: induction of ER stress and the unfolded protein response results in TXNIP upregulation [38,39] and Ca^2+^ release into the cytosol, which can damage mitochondria and lead to ROS accumulation [40]. Mitochondrial damage can also release other stimulators of NLRP3 into the cytosol: cardiolipin and oxidized mtDNA [41]. One more organelle that is implicated in NLRP3 activity is the lysosome: damage of lysosomes is known to release cathepsin B into the cytosol, where it induces NLRP3 activation [42,43]. Linking these together is autophagy, which can remove damaged organelles and thus relieve the cell of inflammatory signals. Indeed, autophagy has been reported multiple times as a negative regulator of NLRP3 [44]. At the post-transcriptional level, numerous miRNAs have been demonstrated to regulate NLRP3 inflammasome; miR-223 [45], miR-7 [46], miR-1929-3p [47] restrains NLRP3 inflammasome. Additionally, extensive studies indicate lncRNAs also have post-transcriptional regulatory effects on NLRP3 inflammasome activation; examples include nuclear enriched abundant transcript 1 (Neat1) [48], MIAT [49], Gm15441 [50], and Platr4 [51]. Certain molecules may bind to compounds in the NLRP3 inflammasome complex or other related molecules to inhibit it and provide negative regulation. Several proved negative regulators are B-cell adapter for phosphoinositide 3-kinase [52], PYRIN domain-only protein 1 [53] and 2 [54], TRIM30 [55], heat shock protein 70 [56], NLR family CARD-containing 3 protein [57] and autophagy [58].

## 4. The Mechanisms of the Action of Melatonin on NLRP3 Inflammasome Inhibition

Melatonin exerts inhibitory function on NLRP3 inflammasome activation through inhibiting or activating several proteins and pathways. NF-κB is a master regulator of the priming phase of NLRP3 inflammasome activation. Melatonin prevents NLRP3 inflammasome activation by inhibiting NF-κB signaling via RORα [59] and silent information regulator 1 (SIRT1)-dependent deacetylation of NF-κB [59]. ROS is a main trigger of NLRP3 inflammasome activation. Growing evidence shows that melatonin reduces levels of TXNIP, leading to suppression of ROS production and NLRP3 activity [60,61]. TXNIP is also mediator of ER stress induced NLRP3 inflammasome activation. Melatonin downregulates ER-induced TXNIP/NLRP3 pathway in LPS-induced endometritis [61]. SIRT1 is a NAD+ dependent deacetylase and a strong regulator of inflammatory, metabolic, and oxidative stressors of cells [62]. Preceding studies reported that sirtuins attenuate NLRP3 inflammasome activation by deacetylating of NLRP3 protein [63], and melatonin increased SIRT1 activity to inhibit the NLRP3 inflammasome [62,64]. Another pathway that melatonin may modulate is Nrf2 which is an antioxidant protein promoting ROS clean-up [17]. It has been documented that melatonin displays protection against NLRP3 inflammasome activity through Nrf2-mediated ROS scavenging and elimination [64,65]. It is well known that autophagy is a negative regulator for NLRP3 activation. Mitophagy is a subtype of autophagy that helps elimination of dysfunctional mitochondria. Melatonin increased the expression of LC3-II/LC3-I and Atg 5 as autophagy markers and Parkin and PINK-1 as mitophagy markers while suppressing NLRP3 inflammasome in the Subarachnoid hemorrhage (SAH) model [59]. Since 3-MA, an autophagy inhibitor, reverses these beneficial effects of melatonin on NLRP3 inflammasome, the results suggest that the effect of melatonin on NLRP3 inflammasome inhibition is dependent on mitophagy induction. The regulatory function of melatonin on inflammasome partly occurs through post-transcriptional mechanisms. Melatonin inhibits NLRP3 inflammasome complex formation by altering the expression of miRNAs and long noncoding RNAs [66,67,68]. Melatonin was tested against pyroptotic cell death in endothelial cells and, it reduced pyroptotic cell death via noncoding RNA MEG3/miR-223/NLRP3 [69]. Furthermore, melatonin mitigated cardiac fibrosis in mice by blocking lncRNA MALAT1/miR-141-mediated NLRP3 inflammasome activation [66]. Melatonin healed radiation induced lung injury both in vivo and in vitro by suppressing miR-30e/NLRP3 axis [68].

## 5. The Effect of Melatonin on NLRP3 Inflammasome Activation in Diseases

The NLRP3 inflammasome is known to have a critical role in the pathogenesis of many diseases and conditions with inflammatory components [70,71,72,73]; and the suppressive effect of melatonin on the NLRP3 inflammasome has been demonstrated in various in vitro and in vivo models of diseases and injuries, which we review below (listed in Tables S1 and S2).

### 5.1. Central and Peripheral Nervous System

It has been shown multiple times that NLRP3 can induce or contribute to neuroinflammation [73,74] in diseases such as Alzheimer’s disease, multiple sclerosis, and traumatic brain injury [70,71,72]. Thus, modulating its activity is an attractive option in the treatment of nervous system conditions. SAH is a deadly form of stroke with high mortality, in which early brain injury and the associated neuroinflammation following the bleeding cause large-scale neuronal damage [75]. It has been reported that inhibiting NLRP3 activity with melatonin can ameliorate brain damage by downregulating NLRP3, ASC, and caspase-1 levels [76,77,78]. Findings from the rat SAH model suggest that melatonin inhibits NLRP3 activation by a mitophagy-mediated reduction in ROS [77]. Inhibition of the NLRP3 inflammasome rescues neurons and oligodendrocytes from inflammasome-mediated apoptotic cell death [76,79]. Melatonin also diminishes trauma-induced NLRP3 activity in spinal cord injury (SCI) by downregulation of TLR4/NF-κB and NOX2/TXNIP [80,81] and traumatic brain injury (TBI) by NF-κB inhibition, prevention of HMGB1 nuclear export, and reduction in necroptosis through A20 [82]. NLRP3 activation is also implicated in animal models of depression induced via stress or LPS challenge [83]. A recent study by our team has shown that melatonin injection inhibits LPS-induced depressive-like behavior in mice by downregulating NLRP3 activation in the brain [64]. The same study further demonstrated that Nrf2/Sirt1 pathway has a vital role in these effects of melatonin in the N9 mouse microglial cell line. One effect of melatonin in the nervous system is preventing morphine-induced analgesic tolerance [84]. A recent mouse study reveals that morphine-induced NLRP3 activation in the brain is responsible for morphine tolerance, and melatonin pretreatment prevents this activation by eliminating ROS and preventing cathepsin B release [85]. 

### 5.2. Sepsis-Induced Inflammasome Activation

Being a part of the innate immune system, the NLRP3 inflammasome contributes to the response against sepsis, sometimes with deleterious effects. For example, NLRP3 deficient mice were observed to have reduced bacterial load, improved neutrophil phagocytosis [86], and better survival rates [87]. Several sepsis studies investigate the interaction of melatonin with NLRP3 in mouse hearts, where sepsis activates the NLRP3 inflammasome and melatonin injection suppresses this activity, confirmed by a decrease in the levels of mature caspase-1 and IL-1β, NLRP3, and ASC [59,88,89,90]. The identified mechanism of melatonin in these studies is the inhibition of NF-κB nuclear translocation through SIRT1-mediated deacetylation on lysine residue 310, which is consequent to a rise in expression of RORα, Clock, Bmal1, NAMPT, and SIRT1 [59]. Another sepsis study finds that melatonin treatment reverses NLRP3 activation in kidneys of septic rats, coupled with a melatonin-induced rise in mitochondrial superoxide dismutase (SOD2), suggesting that suppression of excess ROS might play a role in melatonin’s effect on NLRP3 inflammasome activation [91].

### 5.3. Cardiovascular and Metabolic Diseases

Inflammasomes have a key role in the development of cardiovascular disease [92], and cholesterol can activate the NLRP3 inflammasome in atherosclerotic lesions, mainly via IL-1β release [93,94]. Intra-gastric or intraperitoneal administration of melatonin reduces the size of atherosclerotic lesions, downregulates expression of NLRP3 inflammasome components, suppresses pyroptosis and NF-κB activity in aortas, and reduces serum IL-1β levels of high-fat diet-fed mice [69,95]. Melatonin exerts these effects through upregulating miR-223 and downregulating the lncRNA MEG3 [69]. Melatonin was proven to be alleviating cardiotoxicity via decreasing NLRP3 inflammasome by restraining oxidative stress [96]. Additionally, melatonin upregulates SIRT3 activity and promotes mitophagy/autophagy in atherosclerotic aortas and macrophages [95]. The “metabolic syndrome” diseases type 2 diabetes and obesity, due to their chronic inflammatory nature [97,98], are associated with NLRP3 inflammasome activation [99]. NLRP3, ASC, caspase-1, or IL-1β deficiency improves insulin sensitivity and glucose tolerance in mouse models of insulin resistance [37,100,101]. In streptozotocin-induced diabetes, intra-gastric melatonin improves the microstructure of the myocardium, restores cardiac function [66], and prevents neuronal cell death [67]. Additionally, it prevents a rise in protein levels of NLRP3, caspase-1, and IL-1β in hearts [66] and brains [67] of diabetic mice. Furthermore, in primary cardiac fibroblasts exposed to a high glucose medium, melatonin modulates NLRP3 by altering levels of two antagonistic non-coding RNAs: miR-141 and lncRNA MALAT1 [66]. Melatonin can be beneficial in treating diabetic wounds since it prevents a high glucose medium-induced rise in levels of NLRP3 inflammasome components in rat primary keratinocytes [1]. Regarding obesity, it is observed that expressions of caspase-1 and IL-1β, as well as a caspase-1 activity, are markedly increased in white adipose tissue of diet- or genetically-induced obese mice [100,102,103]. Caspase-1 also appears to be mediating macrophage influx into white adipose tissue [100]. Additionally, knockouts of NLRP3, ASC, or caspase-1 protect mice from diet-induced obesity [100]. Continuous intraperitoneal melatonin treatment in diet-induced obese mice reduces levels of NLRP3 inflammasome components in white adipose tissue via suppression of NF-κB signaling [104]. In leptin-deficient obese mice, melatonin ingestion reduces hypertrophy, inflammatory cell infiltration, and NLRP3 levels in cardiac tissue through activating the metabolic regulators SIRT1, Nrf2, and AMPK [105].

### 5.4. Respiratory Diseases

Activation of the NLRP3 inflammasome is also present in disease, injury, and inflammation in the respiratory system [106]. For example, asbestos and silica particles, inhalation of which can result in pulmonary fibrosis, can trigger NLRP3 activation in THP1 cells [107] and primary normal human bronchial epithelial cells [108]. LPS-induced acute lung injury (ALI) in mice can trigger the NLRP3 inflammasome in lung tissue [109,110]. The release of neutrophil-derived histones into the bronchoalveolar lavage fluid (BALF) is implicated in the activation of NLRP3 in this model [110]. In asthma, symptomatic patients’ sputum [111] and BALF [112] contain higher levels of IL-1β than asymptomatic patients, implying inflammasome activation. Ovalbumin administration in mice (a common asthma model) is reported to exhibit its effects via NLRP3 activation and IL-1β secretion [113]. Chronic obstructive pulmonary disease (COPD), a disease characterized by progressive airflow obstruction, emphysema, and inflammation within the lungs, has been associated multiple times with NLRP3 inflammasome [106,114]. Mouse COPD models and COPD patients have higher IL-1β in their BALF or sputum [115,116,117], and NLRP3-deficient mice are resistant to smoke-induced COPD [115]. Melatonin’s protective effect is demonstrated in an LPS-induced ALI model, where melatonin administration reduces lung damage, lowers IL-1β and caspase-1 levels, and decreases leukocyte numbers in BALF of LPS-challenged mice [118]. A portion of melatonin’s protective effect in this study can be attributed to its inhibition of histone release within the lung [118]. In animal models of cigarette smoke-induced COPD, melatonin reverses many of the deleterious changes caused by COPD including functional decline and deformation in lungs, rise in BALF IL-1β levels, and elevated protein levels of NLRP3 and mature caspase-1 in lung tissue [62,119]. The protective effects of melatonin in COPD appear to be mediated by SIRT1 activity [62], reduction of TXNIP levels by reduction of ER stress, and a drop in ROS due to improved mitophagy [119]. In ovalbumin-induced airway inflammation in mice, intraperitoneal (IP) melatonin reduces the NLRP3 activation in lungs. Fei et al. showed that melatonin reverses the elevated leukocyte count and IL-1β and IL-18 levels in BALF and prevents an increase of NLRP3 and mature caspase-1 levels in lung tissue [120,121]. Lung expression of TLR2, implicated in asthma, is also increased with ovalbumin and suppressed with melatonin [120]. 

### 5.5. Melatonin on NLRP3 Inflammasome Activation in COVID-19 

A global outbreak, coronavirus disease 2019 (COVID-19), caused by severe acute respiratory syndrome coronavirus 2 (SARS-CoV-2), emerged in 2019, and it has not been completely dealt with yet. Since then, researchers across the world immediately started to investigate the underlying pathological mechanisms of SARS-CoV-2. Recent findings suggest NLRP3 inflammasome dysregulation plays a role in coronavirus infection disease COVID-19 in humans [122]. It has been demonstrated that SARS-CoV viroporin 3a protein [123], SARS-CoV ORF3a protein [124], SARS unique domain of SARS CoV [125], and SARS-CoV E protein [126] activate the NLRP3 inflammasome and, subsequently, IL-1β expression is highly upregulated. Cytokine storm, a hyperactivated immune response, is a crucial part of severe COVID-19 pathogenesis which may lead to acute lung injury, acute respiratory distress syndrome, pneumonia, or death [127,128]. Moreover, cytokine storm was shown to be activated through NLRP3 inflammasome overactivation in Streptococcal toxic shock-like syndrome [129] and implied to be activated the same way during SARS-CoV-2 infection [130]. In many studies, melatonin was indicated to attenuate NLRP3 inflammasome due to its anti-inflammatory, antioxidative, and immunomodulatory effects [14,131]. Taken all the aforementioned information into account, several studies suggest that melatonin might be beneficial in fighting COVID-19 [132,133,134,135]. Although these pieces of evidence implicate that melatonin could be useful in COVID-19 treatment, it has not been thoroughly investigated yet. However, several clinical trials started, and are still ongoing, to test melatonin on COVID-19 [136,137,138,139].

### 5.6. Muscoskeletal Tissue

Intervertebral disc degeneration (IVDD) is a condition in which a loss in the homeostatic balance in vertebral discs leads to extracellular matrix degradation [140,141]. It is known that the production of inflammatory cytokines, including IL-1β, is a primary cause of IVDD [142,143]. Protein levels of NLRP3, mature caspase-1, and IL-1β are increased in discs of severe IVDD patients (compared to mild cases) and in discs of rats with surgically-induced IVDD [144]. This upregulation can be reversed in discs of rats injected with melatonin after the surgery. When human nucleus pulposus (NP) cells from IVDD patients are subjected to IL-1β, NLRP3 inflammasome is activated, while melatonin co-treatment prevents this activation and downregulates NLRP3 inflammasome components via suppression of NF-κB activity and mitochondrial ROS production [144]. TNF-α induced NLRP3 activation in rat primary NP cells reveals that NAMPT has a role in the activation of NLRP3, and melatonin suppresses NAMPT expression through inhibiting NF-κB activity [145]. 

In osteoporosis, inflammatory cytokines such as IL-1β and TNF-α have been implicated as mediators [146,147]. Moreover, the degradation products of bone turnover act as stimulants of NLRP3, and estrogen deficiency-induced osteoporosis is less severe in NLRP3-deficient mice [148,149]. Melatonin’s introduction in osteoporosis can overturn the deleterious effects of NLRP3. IP melatonin injections in ovariectomized osteoporotic mice restored their bone mineral density and improved osteogenic differentiation while suppressing NLRP3 activation in their bone marrow mesenchymal stem cells, apparently via upregulating β-catenin signaling [150], a negative regulator of NLRP3 activity [151].

### 5.7. Gastrointestinal Tissue

Inflammatory regulation must be strict in gastrointestinal tissue as microbes and agents in food could be dangerous to the gastrointestinal tract. NLRP3 expression is elevated in lesion cells of patients with oral lichenoid lesions [152] and oral epithelial tissue of periodontitis patients [153]. In a study of radiation-induced oral mucositis in rats, melatonin administered as an oral gel restores mitochondrial functionality, prevents apoptosis, and inhibits NF-κB and NLRP3 activation in irradiated tongue epithelial cells [154]. Various deleterious effects of excess NLRP3 activity have also been shown in the liver [155]. Mice with constitutively active NLRP3 have widespread pyroptosis, fibrosis, and elevated inflammatory marker expression (IL-1β, TNF-α, caspase-1) in the liver [156]. In mouse models of alcoholic liver disease, alcohol induces NLRP3 activation, and the consequent IL-1β secretion leads to liver inflammation, steatosis [157,158]. The non-alcoholic fatty liver disease involves activation of NLRP3, as shown in mouse models [159,160] and patients [159]. Furthermore, cadmium-induced liver injury in mice results in NF-κB activation and a concomitant rise in NLRP3 expression [161]. Daily IP melatonin injections in another cadmium-induced liver injury model decrease ALT and AST levels—implying restoration of liver function—and IL-1β levels in serum, attenuates hepatocyte death, reduces protein levels of NLRP3 and TXNIP, ROS levels, caspase-1 activity, and TXNIP-NLRP3 interaction in liver tissue of cadmium-administered mice [60]. In the intestines, inflammatory bowel disease (IBD) is a major detrimental disorder. However, given the colon’s abundance in a microorganism, the contribution of NLRP3 activity to IBD progression can vary; and it has been shown to have both pathogenic and protective effects in IBD [162,163,164,165]. NLRP3 could be a mediator of intestinal injury induced by methamphetamine [166]. In a rat model of radiation-induced injury, daily oral application of melatonin restored mitochondrial function, decreased NLRP3 levels and activation, reduced protein levels, and nuclear translocation of NF-κB in small intestines of irradiated rats [167]. 

### 5.8. Other Diseases

Systemic lupus erythematosus is a chronic autoimmune disease with uncertain cause and can present itself in various organs [168]. NLRP3 activation has been identified in lupus by measuring patients’ peripheral blood mononuclear cells [169]. Mouse studies of lupus nephritis, the manifestation of lupus in kidneys, also indicates NLRP3 activation [170,171]. A study of pristane-induced lupus nephritis in mice shows that ingested melatonin restores SIRT1 and Nrf2 protein levels, reduces NF-κB and NLRP3 levels, and reverses lesions and morphological alterations in kidneys of pristane-administered mice [172]. NLRP3 levels are reported to be increased in uterine tissue in endometritis models [173,174]. In a mouse LPS-induced endometritis model, melatonin reverses neutrophil infiltration and the rise in ROS, ER stress, and protein levels of NLRP3 inflammasome components in endometrial tissue through inhibiting NF-κB and TXNIP and upregulating AMPK [61]. 

## 6. In Vitro Studies of NLRP3 Inflammasome and Melatonin Interactions

In addition to animal models of disease, the suppressive effect of melatonin on NLRP3 inflammasome activation has also been shown in immune and non-immune cells. A study on the radiation-induced NLRP3 activation model in RAW 264.7 macrophages reveals that melatonin reverses the rise in protein levels of NLRP3, ASC, and secreted IL-1β, suppresses ROS formation, and restores the expression of miR-30e that targets NLRP3 mRNA [68]. In in vitro, LPS plus histone stimulated NLRP3 activation model with mouse primary peritoneal macrophages, melatonin prevents—in a dose-dependent manner—the rise in levels of mature, but not pro-forms of IL-1β and caspase-1; indicating that it inhibits the activation but not the priming of the NLRP3 inflammasome [118]. Interestingly, unlike most melatonin studies that examine ROS levels, melatonin here does not prevent a rise in mitochondrial ROS induced by LPS and histones [118]. In a thrombin-induced inflammation model in BV2 microglia, melatonin treatment again suppresses NLRP3 levels and activation by downregulating ROS [175]. Melatonin suppresses NLRP3 inflammasome activation in non-immune cells as well as in immune cells. In human neuroblastoma (SH-SY5Y) and astrocytoma (U251) cell lines, LPS-induced NLRP3 activation can be suppressed with melatonin treatment [176]. Melatonin also inhibited NLRP3 inflammasome activation induced by cigarette smoke extract in human umbilical vein endothelial cells (HUVECs) [177]. Melatonin treatment in mouse primary adipocytes challenged with LPS protects them from pyroptosis by reducing NF-κB signaling, inhibiting NLRP3 inflammasome activation, and downregulating GSDMD mRNA levels [104]. Melatonin and HIS, a melatonin-derivative, were proven to be protective against LPS-induced NLRP3 inflammasome activation in mouse RAW 264.7 macrophages and BMDMs by attenuation of STAT1 and IRF3 phosphorylation [178].

## 7. Conclusions

**Figure 1:**
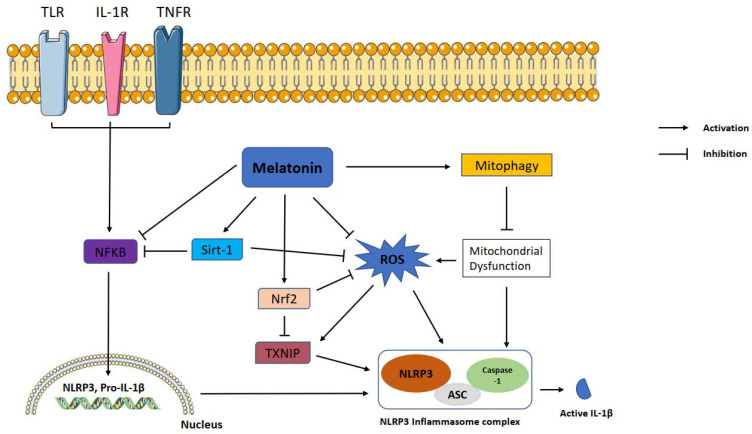
Representation of melatonin effects on NLRP3 inflammasome activation. Melatonin shows ameliorative effects on NLRP3 inflammasome activation via interacting with certain signaling pathways. NLRP3: NLR family pyrin domain containing 3; ASC: apoptotic-associated speck-like protein containing a caspase recruitment domain; ROS: reactive oxygen species; TXNIP: thioredoxin-interacting protein; Nrf2: nuclear factor erythroid 2-related factor 2; Sirt-1: sirtuin 1, IL-1β: interleukin 1β; NF-κB: nuclear factor kappa B.

Over the decades, increasing evidence has demonstrated that melatonin exerts a modulatory effect on the inflammatory response. Figure 1 proposes the pathology associated with melatonin, NLRP3 inflammasome and, essential protective mechanisms. Therefore, melatonin is a promising molecule for the treatment of inflammation-related disorders. It can inhibit the excessive inflammatory response of immune cells and reduce the production of proinflammatory molecules and free radicals through activation or inhibition of different signaling pathways. Melatonin demonstrates anti-inflammatory effects either via direct antioxidant action or by activating receptors. Previous in vitro and in vivo studies established that melatonin suppresses the NLRP3 inflammasome mainly by inhibition of NF-κB signaling, activation of SIRT1 and Nrf2 pathways, and inhibition of ROS production. Recent studies identify a novel mechanism of melatonin. This novel mechanism modulates the expression of non-coding RNAs (miRNAs and LncRNAs) that participate in NLRP3 inhibition. Non-coding RNAs take roles in epigenetic post-transcriptional gene expression control mechanisms. Non-coding RNA modulation may be the mechanism underlying melatonin’s pleiotropic effects due to the ability of non-coding RNAs to target hundreds of genes. The results of preclinical studies cannot be translated to the clinic directly. It is still necessary for future clinical trials and cohort studies to explore the safety and efficacy of melatonin in different age groups. Additionally, a combination of other agents, administration routes, and loading into drug carriers should be considered for future studies.

## Data Availability

Data sharing not applicable to this article as no datasets were generated or analyzed during the current study.

## Supplementary Materials

The following are available online at https://www.mdpi.com/article/10.3390/antiox10071020/s1, Table S1: In vivo studies of melatonin involving NLRP3, Table S2: In vitro studies of melatonin involving NLRP3.
