# Supplementary material for: The Role of Melatonin on NLRP3 Inflammasome Activation in Diseases
Source: Antioxidants (Basel). 2021 Jun 24;10(7):1020. doi: 10.3390/antiox10071020 (PMC8300798; doi:10.3390/antiox10071020)
Supplement: Supplementary file 1 [file antioxidants-10-01020-s001.zip › antioxidants-1209992-supplementary.pdf]

Supplementary Table S1. *In vivo* studies of Melatonin involving NLRP3.

	Organism	Model	Melatonin (MLT) Administration	Inflammasome Parameters Studied	Findings about Melatonin	Mechanism of Inflammasome Suppression	Reference
Central Nervous System	Mouse	Subarachnoid hemorrhage (SAH) via surgery	150 mg/kg, IP, 2 h post-surgery	NLRP3, ASC, caspase-12, IL-1β	Improves neuron and brain health; attenuates protein levels of NLRP3, ASC, caspase-1, IL-1β, IL-6, Bim; increases expression of Bcl2 in brain tissue of SAH mice.	N/A	[78]
	Rat	SAH via surgery	150 mg/kg, IP, 2 h after SAH	NLRP3, ASC, caspase-1	Upregulates mitophagy and autophagy proteins; suppresses ROS generation and NLRP3 activation in brain tissue of SAH mice.	Autophagy/mitophagy	[77]
	Rat	Spinal cord injury (SCI)	12.5 mg/kg/day, IP, for 10 consecutive days after SCI	NLRP3, caspase-1, IL-1β	Improves locomotion functions; restores neuron numbers; suppresses NLRP3, caspase-1 and IL-1β protein levels in spinal cord.	N/A	[176]
	Mouse	LPS-induced depressive like behavior (DLB)	500 mg/kg, IP, 4 doses with 6 h intervals, starting 2 h before LPS	NLRP3, caspase-1, IL-18, IL-1β	Ameliorates DLB; decreases NLRP3, caspase-1, IL-18, IL-1β protein levels and active microglia in mouse hippocampi.	N/A	[64]
	Rat	Controlled cortical impact (CCI) injury on rat brain	20 mg/kg, IP, 1 h before CCI	NLRP3, IL-1β	Suppresses NLRP3 activation, necroptosis, NF-kB activity, RAGE, IL-1β and IL-18 levels; prevents nuclear export of HMGB1 in brains of CCI rats.	A20/TNFAIP3-mediated necroptosis inhibition, inhibition of HMGB1 release, NF-kB inhibition	[82]
	Rat	SCI	10 mg/kg neck injection (SC) after SCI, every 12 h until 72 h	NLRP3, ASC, caspase-1, IL-1β, IL-18	Decreases the loss in locomotor activity; reduces mRNA expression of NLRP3, caspase-1, TLR4, NF-kB, NOX2, TXNIP; reverses the rise in protein levels of NLRP1, AIM2, ASC, caspase-1, IL-1β, IL-18 in spinal cord tissue of SCI rats.	Downregulation of TXNIP and NF-kB expression	[81]
	Mouse	SAH via surgery	50-300 mg/kg IP injection, 15 min after SAH induction	NLRP3, caspase-1, IL-1β	Reverses loss of neurological function and myelin basic protein content; reverses the rise in NLRP3, caspase-1, IL-1β protein levels; reduces accumulation of amyloid precursor protein in brains of SAH mice.	N/A	[76]

	Rat	SCI	10 mg/kg SC injection, every 12 h for 72 h post-SCI	NLRP3, ASC, caspase-1, IL-1β, IL-18	Ameliorates locomotor activity; reverses the rising mRNA and protein levels of NLRP3, ASC and caspase-1; reverses the rise in secreted IL-1β and IL-18 in spinal cord tissue of SCI rats (effects are vertebra-dependent).	N/A	[80]
	Mouse	Hot-plate, tail-flick, acetic acid pain tests on morphine-treated mice	0.5 mg/kg IP injection 30 min before morphine, daily for 7 days	NLRP3, ASC, caspase-1, IL-1β	Inhibits analgesic tolerance to morphine; reverses the rise in NLRP3, ASC, IL-1β, caspase-1, Cathepsin B expression in brains; reverses the rise in IL-1β levels in serum of morphine-treated mice. (NLRP3$^{-/-}$ mice are more resistant to both pain and morphine tolerance)	N/A	[85]
Sepsis	Mouse	Sepsis via Cecal ligation and puncture (CLP) on wildtype and RORα knockout mice	30 mg/kg in 3 or 4 doses: 1 intraperitoneal (IP) dose pre-, 2-3 subcutaneous (SC) doses post-surgery	NLRP3, IL-1β	Disrupts NF-kB deacetylation; suppresses NLRP3 activation in heart tissue of septic mice.	RORα and SIRT1 dependent NF-kB inhibition (deacetylation)	[59]
	Mouse	Sepsis via CLP	30 mg/kg in 3 doses: 1 IP dose pre-, 2 SC doses post-surgery	NLRP3, ASC, caspase-1	Blunts sepsis-induced NLRP3 activation in heart tissue of septic mice.	RORα, Clock/Bmal1 and SIRT1 dependent NF-kB inhibition (deacetylation)	[89]
	Mouse	Sepsis via CLP	30 mg/kg in 3 doses: 1 IP dose pre-, 2 SC doses post-surgery	NLRP3, caspase-1, IL-1β	Improves cardiomyocyte morphology; lowers NLRP3, caspase-1, IL-1β protein levels and NF-kB nuclear translocation; increases Clock, Bmal1, Sirt1 and Nampt mRNA expression in heart tissue of septic mice.	NF-kB inhibition through Clock/Bmal1/Sirt1 pathway	[88]
	Rat	Sepsis via CLP	150 mg/kg in 3 doses: 1 IP dose pre-, 2 SC doses post-surgery	NLRP3, ASC, caspase-1, IL-1β, IL-18	Reverses tissue damage, the reduction in SOD2, and the rise in NLRP3, caspase-1, PINK1, IL-18, IL-1β, IL-6 protein levels in kidneys of septic rats.	ROS downregulation	[91]

	Mouse	Sepsis via CLP on WT and $NLRP3^{-/-}$ mice	30 mg/kg in 3 doses: 1 IP dose pre-, 2 SC doses post-surgery	NLRP3 (no MLT-NLRP3 interaction studied)	Promotes Nrf2 expression and nuclear translocation; improves mitochondrial respiration rates; reverses the pro-apoptotic profile of cells (as measured by Bax, Bcl2, p53 and caspases); suppresses the rise in autophagy and mitophagy associated proteins in heart tissues of septic mice. Effects on oxidative stress protection enzymes are variable. Effects are less pronounced in $NLRP3^{-/-}$ mice.	N/A	[90]
Cardiovascular and Metabolic Diseases	Mouse	IP LPS-challenge; Diet-induced obesity	20 mg/kg/day, IP, for 14 days after LPS or in obese mice	NLRP3, ASC, caspase-1, IL-1β, GSDMD	Reduces serum IL-1β, white adipose tissue mRNA and protein expression of NLRP3, ASC, pro-caspase-1, GSDMD in obese, LPS-challenged, or vehicle mice.	NF-kB inhibition	[104]
	Mouse	High fat diet-induced atherosclerosis in ApoE-/- mice	10 mg/kg/day, via gastric gavage, for 12 weeks	NLRP3, ASC, caspase-1, IL-1β, IL-18, GSDMD	Alleviates atherosclerosis; reduces IL-1β and IL-18 levels in serum; downregulates NLRP3, ASC, caspase-1, IL-1β, IL-18, GSDMD mRNA and MEG3 lncRNA and restores miR-223 in mice aortas.	NF-kB inhibition, MEG3 decrease-induced miR-223 upregulation	[69]
	Mouse	High fat diet-induced atherosclerosis in ApoE-/- mice	20 mg/kg/day, IP, for 28 days	NLRP3, ASC, caspase-1	Slows lesion progression; reduces NLRP3, caspase-1 and mature IL-1β protein levels; increases Sirt3 activity and mitophagy in atherosclerotic lesions; decreases IL-1β in serum.	Sirt3 activation, autophagy/mitophagy	[95]
	Rat	Smoke exposed rats	10 mg/kg/day, IP, for 2 weeks (coadministration)	NLRP3, ASC, caspase-1, IL-1β, IL-18	Reduces protein levels of NLRP3, ASC, caspase-1, IL-1β, IL-18; lowers ROS and malondialdehyde (MDA) production; reverses the fall in SOD in carotid arteries; decreases IL-1β and IL-18 in serum.	ROS downregulation	[177]

	Mouse	Streptozotocin-induced diabetes mellitus (DM)	10 mg/kg/day, via gastric gavage, for 8 weeks	NLRP3, ASC, caspase-1, IL-1β, and IL-18	Restores cardiac function; reduces fibrosis, collagen production and TGF-β1, p-Smad2, p-Smad3, NLRP3, ASC, caspase-1, IL-1β, IL-18 protein levels; reverses the rise in lncRNA MALAT1 and the decline in miR-141 in cardiac tissue of DM mice.	Upregulation of miR-141 (targets NLRP3), downregulation of MALAT1 (targets miR-141)	[66]
	Mouse	Leptin deficient obese mice	100 mg/kg/day, in drinking water, for 8 weeks	NLRP3	Restores AMPK, SIRT1, NRF2 activity; restores PGC-1α, SIRT1, NRF2, HO-1 protein levels; reverses the rise in NLRP3, TNF-α, IL-6 protein levels and inflammatory leukocyte infiltration in hearts; decreases TNF-α, IL-6 levels in serum of obese mice.	AMPK/SIRT1/NRF2 activation	[105]
	Mouse	Streptozotocin-induced DM	10 mg/kg/day, via gastric gavage for 8 weeks	NLRP3, caspase-1, IL-1β, GSDMD	Increases neuron numbers; reverses the rise in NLRP3, IL-1β, cleaved GSDMD and caspase-1; suppresses the rise in autophagy-associated proteins in brains of DM mice.	N/A	[67]
	Rat	Aflatoxin B1 induced cardiotoxicity	5 mg/kg/day, via gastric gavage, for 6 weeks	NLRP3, ASC, caspase-1, IL-1β	Restores SOD levels, reduces NLRP3, ASC, caspase-1, IL-1β protein levels, reverses the rise in NF-kB activation and ROS in myocardium of aflatoxin-treated rats.	NF-kB inhibition, ROS downregulation	[96]
Respiratory System	Mouse	LPS-induced acute lung injury (ALI)	30 mg/kg, IP (1 dose before, 2 doses after ALI) or intratracheal (2 doses after ALI).	caspase-1, IL-1β	Reduces lung damage; decreases leukocyte number, free histones, caspase-1 and IL-1β in bronchoalveolar lavage fluid (BALF).	Inhibiting histone release (suggested)	[118]
	Rat	LPS and cigarette smoke induced COPD model	10 mg/kg/day, IP, for 28 days	NLRP3, ASC, caspase-1, IL-1β	Improves lung function; reduces IL-1β and inflammatory cells in BALF; decreases NLRP3, ASC, caspase-1 and increases SIRT1 protein levels in lung tissue. Anti-inflammasome effects are lost with SIRT1 inhibition.	SIRT1 upregulation	[62]
	Mouse	Radiation induced lung injury	1 mg/day, IP, for 7 days post irradiation	IL-1β	Protects against lung damage; restores levels of SOD in lung tissue; reverses IL-1β increase in BALF.	N/A	[68]

	Mouse	Ovalbumin induced airway inflammation	10 mg/kg, IP, 1 h before ovalbumin	NLRP3, caspase-1, IL-1β, IL-18	Ameliorates airway inflammation, lowers leukocyte count and Th2-associated cytokines in BALF, reduces NLRP3 activation in lungs. CpG-ODNs suppress inflammation, and boost melatonin biosynthesis.	N/A	[121]
	Mouse	Ovalbumin induced airway inflammation in WT and TLR2$^{-/-}$ mice	10 mg/kg, IP, 1 h before ovalbumin	NLRP3, caspase-1, IL-1β, IL-18	Improves tissue morphology; reduces TLR2 expression, leukocyte infiltration and NLRP3 activation, and boosts melatonin synthesis in lungs. Airway inflammation decreases melatonin synthesis in lungs; lack of TLR2 receptors rescues this effect.	TLR2 downregulation	[120]
	Mouse	Cigarette smoke induced COPD model	2.5-20 mg/kg IP injection 1 h before smoke for 4 weeks	NLRP3, caspase-1, IL-1β, IL-18	Reverses cell damage, inflammatory cell number, alveolar deformation and MPO activity; reduces ROS, MDA and reverses the fall in antioxidant enzymes; returns mitochondrial membrane potential closer to normal; reduces expression of TNF-α, IL-1β, IL-18 in BALF; reverses the rise in levels of ER stress related proteins, TXNIP, NLRP3, caspase-1 and IL-1β; raises levels of antioxidant and mitophagy-promoting proteins in lungs of smoke-exposed mice.	ROS downregulation, reduction of TXNIP levels (by reduction of ER stress), mitophagy	[119]
Muscoskeletal Tissue	Mouse	Ovariectomy-induced osteoporosis	10 or 50 mg/kg/day, IP, for 8 weeks	NLRP3, ASC, caspase-1, IL-1β, IL-18	Restores bone mineral density; improves osteogenic differentiation in bone marrow mesenchymal stem cells (BMSCs); decreases protein levels of NLRP3, ASC, caspase-1, IL-1β in femoral bone and BMSCs.	Wnt/β-catenin pathway activation	[150]
	Rat	Puncture-induced intervertebral disc degeneration (IVDD)	30 mg/kg/week, IP, after surgery	NLRP3, ASC, caspase-1, IL-1β	Delays the progression of IVDD; suppresses NLRP3 inflammasome activation in vertebral tissue.	N/A	[144]

Other Organs and Tissues	Rat	Radiation-induced oral mucositis	45 mg/day, IP or oral gel, for 21 days	NLRP3, ASC, caspase-1, IL-1β	Restores mitochondrial functionality; prevents apoptosis, NF-kB and NLRP3 activation in tongue tissue (when given as oral gel).	NF-kB inhibition	[154]
	Mouse	Cadmium-induced liver injury	10 mg/kg/day, IP, for 3 days before Cadmium	NLRP3, caspase-1, IL-1β	Decreases serum ALT/AST levels; attenuates hepatocyte death, NLRP3 activation, ROS and TXNIP expression in liver tissue.	Inhibition of TXNIP-NLRP3 interaction, ROS downregulation	[60]
	Rat	Radiation induced intestine injury	45 mg/day, oral application (gel), 2 days before the first irradiation, until 14 days after the last irradiation	NLRP3, caspase-1, IL-1β	Restores expression of mitochondrial proteins; suppresses NLRP3 activation, NF-kB expression and translocation, and apoptosis in rat small intestine.	NF-kB inhibition	[167]
	Mouse	LPS-induced endometritis	5, 10 or 20 mg/kg, IP, 1 h before LPS injection and 25 h before sacrifice	NLRP3, ASC, caspase-1, IL-1β	Reduces myeloperoxidase (MPO) activity, NF-kB activation and ROS; reverses the increase in TXNIP, NLRP3, ASC, caspase-1, IL-1β and ER stress protein levels; increases AMPK activity in uterus tissue.	Suppression of TXNIP-NLRP3 interaction and ER stress, AMPK activation	[61]
	Mouse	Pristane-induced Lupus Nephritis	10 mg/kg/day in drinking water, after pristane delivery, for 6 months	NLRP3	Restores kidney morphology; reduces TNF-α, NF-kB, iNOS, NLRP3 protein levels and restores SIRT1, NRF2 protein levels in the kidney.	SIRT1/NRF2 increase	[172]

Supplementary Table S2. *In vitro* studies of Melatonin involving NLRP3

Topic	Cell Line	Inflammasome Inducer	Melatonin Administration	Inflammasome Parameters Studied	Findings about Melatonin	Mechanism of Inflammasome Suppression	Reference
Central Nervous System	Human SH-SY5Y neuronal and U251 glial cells	LPS-induced NLRP3 activation	10 μM, co-treatment	NLRP3, caspase-1, IL-1β	Suppresses NLRP3, IL-1β and caspase-1 protein and mRNA levels.	N/A	[176]
	Mouse N9 microglia	LPS+ATP induced NLRP3 activation	500 μM, 1 h pretreatment + co-treatment with LPS & ATP	NLRP3, ASC, caspase-1, IL-18, IL-1β, GSDMD	Decreases NLRP3, caspase-1 protein levels, ASC speck formation, IL-18 & IL-1β secretion, mtROS production, GSDMD cleavage, pyroptosis and NF-kB activation; increases SIRT1 and NRF2 activity Anti-inflammasome effects are reversed with SIRT1 or NRF2 inhibition.	NF-kB inhibition, ROS downregulation, SIRT1/NRF2 activity	[64]
	Mouse primary microglia	Inflammasome activation by morphine + nigericin or LPS + nigericin	200 μM for 30 min before LPS or morphine	NLRP3, ASC, caspase-1, IL-1β, GSDMD	Reverses the rise in ROS, protein levels of NLRP3, ASC, mature caspase-1 and IL-1β, Cathepsin B in morphine- or LPS-induced microglia.	ROS downregulation, inhibition of Cathepsin B release	[85]
Cardiovascular and Metabolic Diseases	Rat primary keratinocytes	High-glucose (HG) medium treatment	1 mM, 24 h pre-treatment	NLRP3, ASC, caspase-1, IL-1β	Reduces mRNA and secreted levels of TNF-α, IL-1β, IL-6, IL-8; suppresses ROS, caspase-1 activity, apoptosis, expression of NLRP3 inflammasome components; restores SOD activity, cell proliferation.	ERK signaling	[1]
	Human Aortic Endothelial Cells (HAECs)	ox-LDL treatment	10 μM for 48 h after ox-LDL	NLRP3, ASC, caspase-1, IL-1β, IL-18, GSDMD	Reverses the increase in MEG3 and decrease in miR-223; lowers protein levels of NLRP3, ASC, caspase-1, IL-1β, IL-18, GSDMD-N.	MEG3 decrease-induced miR-223 upregulation	[69]
	Mouse RAW264.7 macrophages	ox-LDL treatment	10 μM for 24 h, co-treatment	NLRP3, ASC, caspase-1, IL-1β	Decreases NLRP3 inflammasome activation, IL-1β secretion and ROS production;	Sirt3/FOXO3a activation, Autophagy/mitophagy, ROS downregulation	[95]

					increases Sirt3 activity, FOXO3a deacetylation and mitophagy. Changes are reversed with Sirt3 knockdown.		
	Mouse primary cardiac fibroblasts	HG medium treatment	10 μM, 48 h	NLRP3, ASC, caspase-1, IL-1β, IL-18	Reduces collagen production and TGF-β1, p-Smad2, p-Smad3, NLRP3, ASC, caspase-1, IL-1β, IL-18 protein levels; reverses the rise in lncRNA MALAT1 and the decline in miR-14 in HG-treated fibroblasts.	Upregulation of miR-141 (targets NLRP3), downregulation of MALAT1 (targets miR-141)	[66]
	Human SH-SY5Y neuronal cells	HG medium treatment	10 μM for 24 h		Reduces cell death; reverses the rise in NLRP3, IL-1β, cleaved GSDMD and caspase-1; suppresses the rise in autophagy-associated proteins; reverses the fall in miR-214-3p (a caspase-1 targeting miRNA).	miR-214-3p upregulation	[67]
Respiratory System	Human L-132 lung alveolar epithelium cells	CSE administration	0-50 μM 1 h before CSE	NLRP3	Increases cell viability, reduces toxic effects of CSE; reverses the rise in NLRP3 and TXNIP protein expression; reduces ROS levels in CSE-treated cells. (ROS inhibitor NAC increases effects further).	TXNIP and ROS downregulation	[119]
Muscoskeletal Tissue	Rat primary nucleus pulposus cells	TNF-α induced NLRP3 activation	50-1000 nM, 1-24 h	NLRP3, caspase-1, GSDMD	Suppresses NAMPT expression, TNF-α induced NLRP3 activity and matrix degradation (NAMPT expression induces NLRP3 activation).	Suppression of NAMPT/MAPK/NF-kB signalling	[145]
	Human nucleus pulposus cells from intervertebral	IL-1β induced NLRP3 activation	1 mM, 24 h	NLRP3, ASC, caspase-1, IL-1β	Reverses IL-1β induced NLRP3 activation and rise in pro-IL-1β, pro-caspase-1, NLRP3	NF-kB inhibition, ROS downregulation	[144]

	disc degeneration patients				mRNA; reduces NF-kB signaling and mtROS production.		
Other Tissues	Mouse primary hepatocytes	Cadmium-induced NLRP3 activation	1 mM, 2 h pre-treatment	NLRP3, caspase-1, IL-1β	Reduces cadmium-induced cell death, NLRP3 activation and TXNIP-NLRP3 interaction.	Inhibition of TXNIP-NLRP3 interaction, ROS downregulation	[60]
In Vitro General	Mouse RAW 264.7 macrophages and BMDMs	RAW 264.7: LPS BMDMs: LPS priming + ATP or Nigericin	HIS, a MLT-derivative is used. RAW: 40 μM HIS or MLT, 1 h pre-treatment BMDM: 40 μM HIS, 1 h pre-treatment	NLRP3, ASC, caspase-1, IL-1β	HIS is more effective at reversing inflammatory changes than MLT at the same concentration in RAW264.7 cells. HIS inhibits caspase-1 and IL-1β secretion, but not inflammasome complex protein levels in LPS+ATP treated BMDMs.	Attenuation of STAT1 and IRF3 phosphorylation	[178]
	Mouse primary peritoneal macrophages	LPS and histone induced NLRP3 activation	0.1-5 mM, 30 min after 4 h LPS	caspase-1, IL-1β	Suppresses IL-1β and caspase-1 release from cells dose-dependently; does not decrease mtROS.	N/A	[118]
	Mouse primary adipocytes	LPS-induced NLRP3 activation	1 μM for 14 h alone + 10 h with LPS	NLRP3, ASC, caspase-1, IL-1β, GSDMD	Suppresses NF-kB activation, NLRP3 activation, GSDMD mRNA levels and pyroptosis.	NF-kB inhibition	[104]
	Mouse RAW 264.7 macrophages	Radiation-induced NLRP3 activation	500 μM for 12 h after irradiation	NLRP3, ASC, caspase-1, IL-1β, IL-18	Reduces NLRP3, ASC, IL-1β protein levels, ROS and pyroptosis; restores miR-30e expression.	ROS downregulation, miR-30e (targeting NLRP3)	[68]
	Human umbilical vein endothelial cells (HUVECs)	Cigarette smoke extract (CSE)-induced NLRP3 activation	100 μM, 3 h pre-treatment + 24 h co-treatment	NLRP3, ASC, caspase-1, IL-1β, IL-18, GSDMD	Reduces ROS production, cell death and protein levels of NLRP3, ASC, caspase-1, IL-1β, IL-18, GSDMD-N.	ROS downregulation	[177]
	Mouse BV2 microglia and HT22 neuronal cells	BV2: Thrombin-induced inflammation HT22: Medium from induced BV2 cells	2-100 μM (effective dose = 20 μM), 30 min pre-treatment	NLRP3, ASC, caspase-1, IL-1β, IL-18	Reverses the rise in ROS and protein levels of NLRP3, ASC, IL-1β, IL-18 in thrombin-induced BV2 cells. (HT22 cells treated with thrombin + melatonin conditioned	ROS downregulation	[175]

	medium is protected against ROS and apoptosis, compared to those treated with thrombin conditioned medium)	
